# Acknowledgement to Reviewers

**DOI:** 10.1111/bpa.13220

**Published:** 2023-12-10

Each year hundreds of reviewers contribute their expertise to peer review, a process that contributes critically to the quality of the Brain Pathology. The editors at Brain Pathology would like to extend their gratitude to those who have provided their time and energy to review manuscripts for our journal over the last year. We are well aware that our journal can only exist thanks to your concerted efforts to provide concise, accurate, and thoughtful reviews. Below is a list of all of you who completed at least one review, and agreed to have your name published. We also thank those reviewers who choose not to have their names published.

Ahrendsen, Jared

Alexandrescu, Sanda

Auer, Roland

Ayton, Scott

Battini, Jean‐Luc

Bieniek, Kevin

Bockmayr, Michael

Cai, Jinquan

Carare, Roxana

Cullell, N

Del Bigio, Marc

Dudek, Ed

Englert, Benjamin

Evelson, Pablo

Falkenburger, Björn

Ferrer, Isidre

Gilani, Ahmed

Giustetto, Maurizio

Glatzel, Markus

Gu, Yan

Guo, Jifeng

Guzman, Samuel

Han, Lei

Hawkins, Cynthia

Herms, Jochen

Highley, Robin

Horbinski, Craig

Irwin, David

Joseph, Jeffrey

Joutel, Anne

Kovacs, Gabor

Lammens, Martin

Langdon, Kristopher

Leske, Henning

Levine, Adrian

Li, Jianrong

Liu, Ying

Marklund, Niklas

Mechtler, Karl

Meinhardt, Jenny

Munoz, David

Nasrallah, MacLean

Nicolas, Gael

Nordin, Angelica

Piao, Yueshan

Pittella, José

Popa‐Wagner, Aurel

Priller, Josef

Puig, Berta

Reimann, Jens

Rosi, Susanna

Rozemuller, Annemieke

Rushing, Elisabeth

Saleeb, Rola

Satomi, Kaishi

Schulz‐Schaeffer, Walter J

Sepulveda‐Falla, Diego

Shelkovnikova, Tatyana A

Springer, Wolfdieter

Tan, Jieqiong

Troakes, Claire

Vazquez‐Manrique, Rafael

Vinters, Harry

Wakabayashi, Koichi

Wang, Yanjiang

Wang, Yu

Xu, Yuqiao

Yao, Yu

Zhao, Junli

Zheng, Danfeng

Zimmermann, Marina

